# La para-osteo-arthropathie neurogene dans le syndrome de guillain barre: complication rare (à propos d'un cas et revue de la littérature)

**DOI:** 10.11604/pamj.2015.20.245.3143

**Published:** 2015-03-13

**Authors:** Hatim Abid, Mohamed El Idrissi, Mohamed Shimi, Abdelhalim El Ibrahimi, Abdelmajid El Mrini

**Affiliations:** 1 Service de Chirurgie Ostéo-articulaire B4, CHU Hassan II, Fès, Maroc

**Keywords:** Para-ostéo-arthropathie, hanche, syndrome de Guillain-Barré, Para-osteo-arthropathie, hip, Guillain-Barré syndrome

## Abstract

Les para-ostéo-arthropathies neurogènes sont des complications classiques des affections neurologiques centrales, surtout dans les contextes traumatiques. Elles surviennent principalement au voisinage des grosses articulations. Leur physiopathologie exacte reste inconnue malgré de très nombreux travaux et cas rapportés. Il semble que leur survenue au décours d'affections neurologiques périphériques soit exceptionnelle. Nous présentons le cas d'une para-ostéo-arthropathie de hanche bilatérale compliquant un syndrome de Guillain-Barré.

## Introduction

Les para-ostéo-arthropathies neurogènes se définissent comme étant des ossifications ectopiques juxta-articulaires péri ou para-osseuses respectant l'articulation. Leur diagnostic est posé généralement de façon tardive. Elles sont souvent décrites au décours d'affections neurologiques centrales: encéphaliques ou médullaires post traumatiques, et restent exceptionnelles dans les suites d'une neuropathie périphérique. A ce propos, Nous rapportons dans ce travail l'observation d'une patiente présentant des ossifications péri articulaires bilatérales de la hanche, survenues après un syndrome de Guillain – Barrée, avec une revue de la littérature afin de rappeler la physiopathologie de cette maladie qui demeure mal élucidée, et de mettre le point sur ses circonstances de découverte, les moyens diagnostiques et les éventualités thérapeutiques disponibles.

## Patient et observation

Une patiente de 42 ans, sans antécédents pathologiques notables, consultait pour douleur chronique des deux hanches, mécanique, associée à une impotence fonctionnelle d'aggravation progressive, apparues après 40 jours d’évolution d'un syndrome de Guillain-Barré diagnostiqué sur des critères cliniques (tétraplégie flasque), électriques (effondrement de la vitesse de conduction nerveuse aux quatre membres à l’électromyogramme), et biologiques (dissociation albumino cytologique à l’étude du liquide céphalo rachidien), et dont la prise en charge initial avait nécessitée un séjour en réanimation pendant 1 mois pour détresse respiratoire. La malade a été trachéotomisée, mise sous ventilation assistée pendant 3 semaines et perfusée par les immunoglobulines.

**Figure 1:**
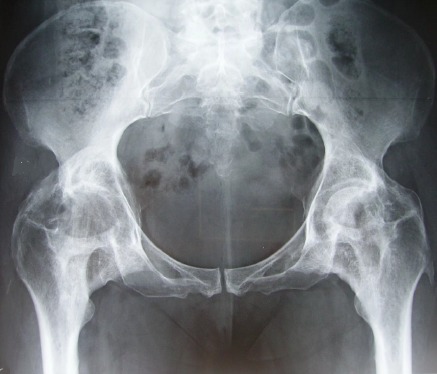
Radiographie standard du bassin de face montrant des ossifications hétérotopiques bilatérales de la hanche

**Figure 2:**
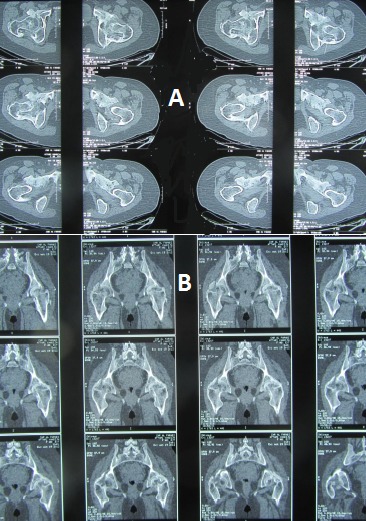
Coupes scanographiques transversales (a) et frontales (b) montrant des ponts osseux antérieurs et postérieurs bilatéraux de la hanche

**Figure 3:**
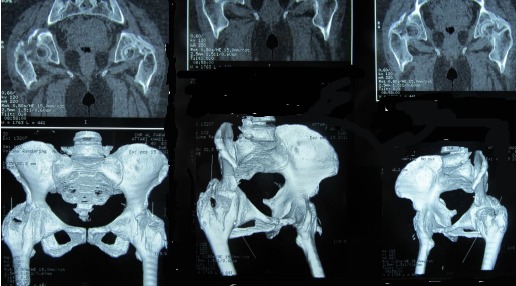
Images scanographiques en reconstruction 3 D des ponts osseux

A l’étape clinique, l'examen révélait une attitude vicieuse des deux hanches en flexion de 20°, irréductible, avec une raideur à la mobilisation et une impossibilité à la marche sans aide, soit un score PMA (Postel Merle d'Aubigné) à 3. La patiente a bénéficié d'un bilan biologique qui n'a pas révélé de syndrome inflammatoire, les facteurs rhumatismaux étaient négatifs alors que les phosphatases alcalines sériques étaient à la limite supérieure normale. Le bilan radiologique a comporté une radiographie standard du bassin de face avec un complément scannographique, mettant en évidence sur la radiographie standard des opacités d'aspect nuageux autour des deux hanches (Figure 1), et sur le scanner, des ponts osseux antérieurs et postérieurs, bilatéraux, pontant l'articulation coxo-fémorale dont l'interligne est respecté (Figure 2 et Figure 3).

## Discussion

La para-ostéo-arthropathie neurogène est une complication rare dans les neuropathies périphériques. Elle se localise aux voisinages des grosses articulations. Dans la littérature, on dénombre 8 cas apparus entre 7 semaines et 17 ans dans les suites d'un syndrome de Guillain –Barrée. Dans ce cadre, Gabi et al. avaient rapporté, entre 1988 et 2001, 4 cas sur une étude qui a intéressé 65 patients suivis pour un syndrome de Guillain-Barré, la hanche était l'articulation touchée et l'atteinte bilatérale concernait deux patients. En 1998, V. Bernard a publié un cas dont l'atteinte siégeait inhabituellement au niveau des deux mains. Puis, sont apparues les publications de Ryu et al en 2008, d'Ohnmar et al en 2010 et de M.Shawgi en 2012, rapportant chacune un cas avec l'atteinte constante de la hanche sans qu'elle soit pour autant bilatérale [1–4]. Notre malade représente le troisième cas d'atteinte simultanée des deux hanches.

Sur le plan physiopathologique, la plupart des auteurs soulèvent l'hypothèse de la transformation des cellules hématopoïétiques placées en situation extra squelettique en cellules ostéogéniques, suite à des arrachements périostés secondaires aux mouvements spastiques chez les comateux et les blessés médullaires [5–7]. En ce qui concerne les neuropathies périphériques, nous n'avons que les résultats expérimentaux de l’étude de Michelsson et Rauschning de 1983, réalisée chez des lapins, et qui a démontré que l'immobilisation seule ne conduit pas à l'apparition des ossifications hétérotopiques péri articulaires, elle doit s'associer nécessairement aux manipulations passives.

D'un point de vue clinique, la plupart des auteurs résume le tableau en des signes inflammatoires locaux à type de rougeur, chaleur et ædème avec réduction progressive de l'amplitude articulaire évoluant vers la raideur [8]. Chez notre patiente, nous avons noté l'absence de cette phase inflammatoire. La douleur était d'emblée d'allure mécanique, déclenchée par la tentative de mobilisation passive et active des deux hanches lors des séances de kinésithérapie et au moment de la marche.

Les phosphatases alcalines, qui représentent l'index prédictif de la synthèse de collagène osseuse, constituent le signe para-clinque le plus précoce, le plus utile et le moins couteux pour la détection des ostéotomes. Sa normalité n'exclut pas le diagnostic comme c'est rapporté dans les travaux de Chantraine et al et ceux de Kim et al, mais subissent invariablement une augmentation précoce [8]. Chez notre patiente, le dosage a été effectué après 45 jours d’évolution de sa neuropathie, le taux retrouvé était à la limite supérieur de la normale, mais avec une augmentation par rapport à une valeur d'admission en réanimation.

L'imagerie permet de faire le diagnostic, d'apprécier la maturation des lésions et de chercher les localisations associées. Sur la radiographie standard, l'aspect varie selon le stade évolutif, de l'image en nuage à un stade précoce, à la zone dense à contours précis au stade de l'ostéome. En 1996, Pistarini et al. ont publié les résultats d'un travail consacré à l'importance de l’échographie dans le diagnostic précoce de la para-ostéoarthropathie neurogène au moment des premiers signes cliniques d'appel. Dans ce cadre, les auteurs soulignent que les premières altérations radiographiques apparaissent à 6 semaines en moyenne après la détection échographique [9]. Pour ce qui est de la scintigraphie, elle est particulièrement importante autant pour le diagnostic précoce que pour la recherche de lésions associées. Elle n'est pas considérée, pour la plupart des auteurs, comme un indicateur sensible concernant la présence des ossifications, car l'hyper fixation peut s'observer lors d'autres affections tel l'arthrose et les fractures associées. La TDM et l'IRM permettent d’établir un bilan lésionnel précis, dans le but de faciliter la décision thérapeutique qui vise de restituer une autonomie fonctionnelle satisfaisante après la stabilisation de l’état neurologique [9, 10].

En terme de prise en charge thérapeutique, La rééducation trouve sa place d'abord à la phase précoce de la maladie en association avec les biphosphonates susceptibles de prévenir l'apparition des ostéotomes selon les résultats de l’étude de Stover et Garland [11, 12]. En postopératoire, la kinésithérapie motrice prend une place considérable, elle doit être douce et poursuivie pendant au moins 3 mois, dans l'espoir de conserver le résultat per opératoire [13]. Toujours à visé préventive, la radiothérapie décrite en 1974 par Parkinson et Coventry semble être efficace sur la survenue des ossifications peri-articulaires si elle est réalisée avant le 4^ème^ jour postopératoire [14, 15].

**Figure 4:**
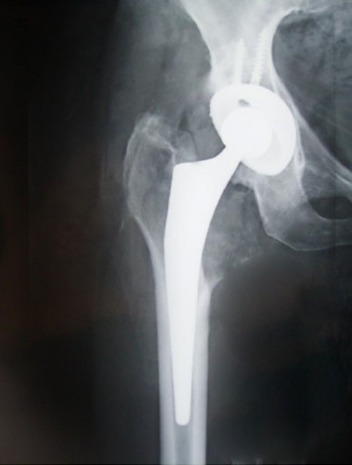
Radiographie de la hanche droite de face après arthroplastie

Au stade de raideur, le traitement chirurgical représente la méthode la plus sure pour restituer au patient une autonomie fonctionnelle satisfaisante. L'intervention pose deux grands problèmes à savoir le choix du délai opératoire et les difficultés techniques. Pour le délai opératoire, il est conditionné par la maturation des ostéotomes, mais surtout par la stabilisation de l’état neurologique comme rapportent la plupart des auteurs, ce délai est de 24 mois pour Rigaux, 34 mois selon Denormandie et 36 mois dans la série de Bouattour. Pour ce qui est des difficultés techniques, elles sont plus importantes au niveau des articulations profondes, tel la hanche, surtout en cas d'ostéome circonférentiel et lorsque l'atteinte est bilatérale. La chirurgie doit concilier une restitution des amplitudes articulaires avec le respect des éléments musculo-tendineux et de la stabilité articulaire. On distingue entre une conduite conservatrice par exérèse des ponts osseux, et une deuxième radicale par arthroplastie. La première méthode est de loin la plus utilisée, la voie d'abord doit être adaptée à la localisation de l'ostéome: voie antérieure, antéro-externe, postérieure ou combinée lorsqu'il s'agit d'une disposition circonférentielle des ostéotomes. Dans ce dernier cas, l'abord est délabrant ce qui expose au risque de nécrose du lambeau cutané intermédiaire, de lésions nerveuses mais surtout d'accidents vasculaires qui peuvent être mortels. Dans ce cadre, Garland et Morallet déplorent des plaies du pédicule fémoral superficiel avec un cas de décès par choc hémorragique dans la série de Bouattour [13]. En ce qui concerne le gain fonctionnel après la résection des ponts osseux, tous les auteurs rapportent une perte de mobilité de 15 à 20 ° entre le moment de l'intervention et l’évaluation post opératoire au recul [13]. La récidive après traitement par résection des ostéotomes est fréquente, elle est souvent précoce et son incidence varie de 6 à 35% selon les séries pour seulement 5 à 15% pour Charnley et Delee après arthroplastie [16]. C'est pour cela, que nous avons opté chez notre patiente, opérée précocement à 6 mois d’évolution de son syndrome de Guillain-Barrée, pour une arthroplastie de la hanche (Figure 4) dans le but de lui procurer le maximum de mobilité sans risque de perte secondaire d'amplitudes, ni de complications cutanées et vasculaires. En post opératoire, les suites étaient simples. À trois mois de recul, la patiente a récupéré son autonomie avec un score PMA actuel à 14. Nous prévoyons d'opérer la malade du coté gauche afin d'optimiser d'avantage notre résultat fonctionnel très encourageant.

## Conclusion

La para ostéo-arthropathie neurogène de la hanche, d'origine multifactorielle, constitue une complication rare dans les neuropathies périphériques. C'est une entité pathologique qu'il faut savoir évoquer devant les premiers symptômes pour prévenir la raideur précoce. La prise en charge doit être multidisciplinaire (réanimateur, kinésithérapeute et orthopédiste). La récupération fonctionnelle ne peut être que meilleure après arthroplastie.
